# Metallic artifact suppression with MAVRIC-SL in magnetic resonance imaging for assessing chronic pain after hip or knee arthroplasty

**DOI:** 10.1590/0100-3984.2023.0026

**Published:** 2023

**Authors:** Gustavo Mota Rios, Carolina Freitas Lins, Milson Carvalho Quadros Junior, Raphaela Lisboa Andrade Nery, Ronald Meira Castro Trindade, Marcos Almeida Matos

**Affiliations:** 1 Delfin Medicina Diagnóstica (Alliança), Salvador, BA, Brazil.; 2 Department of Orthopedics, Hospital Santa Izabel, Salvador, BA, Brazil

**Keywords:** Magnetic resonance imaging, Arthroplasty, Osteolysis, Arthralgia, Prosthesis failure., Ressonância magnética, Artroplastia, Osteólise, Artralgia, Falha de prótese.

## Abstract

**Objective:**

To analyze the association between osteolysis at the prosthesis interfaces, as determined by magnetic resonance imaging (MRI) with multiacquisition variable-resonance image combination selective (MAVRIC-SL) sequences, and clinical severity after knee or hip arthroplasty, as well as to assess interobserver and intraobserver agreement on periprosthetic bone resorption.

**Materials and Methods:**

This was a cross-sectional study of 47 patients (49 joints) under postoperative follow-up after knee or hip arthroplasty, with chronic pain, between March 2019 and August 2020. All of the patients completed the Western Ontario and McMaster Universities Osteoarthritis Index (WOMAC) questionnaire. The component interfaces were evaluated and ordered into two groups: osseointegrated and osteolytic. Nonparametric tests were used.

**Results:**

There were significant differences between the two groups in terms of the mean WOMAC scores: total (*p* = 0.010); stiffness domain (*p* = 0.047); and function (*p* = 0.011) domains. There was substantial interobserver and intraobserver agreement for most analyses of the components.

**Conclusion:**

Periprosthetic osteolysis appears to be associated with clinical complaints of pain in the post-arthroplasty scenario, and MAVRIC-SL provides reproducible assessments. It could prove to be an important tool for orthopedists to use in the evaluation of challenging cases of chronic pain after arthroplasty.

## Abstract (pt)

**Objetivo:**

Analisar associação entre osteólise nas interfaces protéticas por ressonância magnética com sequências MAVRIC-SL e a gravidade clínica dos pacientes submetidos a artroplastias de joelho ou quadril. Determinar concordância intraobservador e interobservador na reabsorção óssea peri-implante.

**Materiais e Métodos:**

Foi realizado estudo transversal entre março de 2019 e agosto de 2020, com 47 pacientes (49 articulações) em seguimento pós-operatório de artroplastias de joelho ou quadril, com dor crônica, que responderam ao questionário WOMAC. As interfaces dos componentes foram avaliadas e definiram dois grupos: osteointegrado e osteólise. Testes não paramétricos foram usados.

**Resultados:**

Houve diferença significativa na média do escore WOMAC entre os grupos (*p* = 0,010), assim como nos domínios rigidez (*p* = 0,047) e função (*p* = 0,011). Houve concordância substancial interobservador e intraobservador para a maioria dos componentes analisados.

**Conclusão:**

Osteólise periprótese parece estar associada com a queixa clínica de dor pós-artroplastia, com avaliação reprodutível pela MAVRIC-SL. Isto pode ser uma importante ferramenta para o ortopedista na avaliação de casos desafiadores de dor crônica pós-artroplastia.

## INTRODUCTION

In cases of advanced osteoarthritis, total hip arthroplasty (THA) and total knee arthroplasty (TKA) are the most effective procedures for the resolution of symptoms and restoration of joint function^**(^1^)**^. Despite the exponential growth in the number of these procedures expected in the coming years, they are not free of complications, often resulting in chronic pain, the clinical differentiation of which is difficult, posing a diagnostic challenge for the orthopedist^**(^2^)**^.

In the diagnostic investigation of prosthesis complications, in addition to the clinical findings, serology, synovial fluid analysis, and radiographs, magnetic resonance imaging (MRI) has gained prominence, although it suffers from magnetic susceptibility artifacts^**(^3^)**^. In addition to conventional two-dimensional fast spin-echo sequences, several MRI techniques, specific to the suppression of metallic artifacts, have been developed in the last decade. Such techniques include slice-encoding for metal artifact correction (SEMAC) and multiacquisition variable-resonance image combination (MAVRIC), both of which are multispectral sequences exciting the overall volume being imaged^**(^4^)**^. A new sequence possessing the advantageous characteristics of the two techniques, presented in 2011, is MAVRIC selective (MAVRIC-SL), which uses frequency-selective excitation with multiple ranges of different frequency bands, minimizing image distortion and providing a high signal-to-noise ratio, together with the Z-selectivity of SEMAC and the view-angle tilting technique. Therefore, compared with conventional two-dimensional fast spin-echo sequences, MAVRIC-SL can reduce metal artifacts significantly and increase diagnostic confidence in patients who have undergone arthroplasty^**(^5^)**^. The use of MAVRIC-SL has allowed a better evaluation of the interfaces with the prosthesis, demonstrating excellent accuracy in the evaluation of osteolysis^**(^6^)**^.

In the last decade, several studies have demonstrated the value of MRI as a complementary method in the context of post-arthroplasty pain^**(^7^,^8^)**^. On MRI, loosening of the prosthesis is characterized by circumferential bone resorption, especially if the resorption is accompanied by displacement, rotation, or sinking^**(^9^)**^. However, those aspects are seen later in the evolution of loosening, which results in greater technical difficulty and reduces the chance of success for revision surgery.

In the cases of patients who develop chronic pain after arthroplasty, without signs of frank loosening or evidence of infection, our hypothesis is that areas of resorption are implicated in clinical worsening. In the literature, data on the analysis of periprosthetic resorption versus the clinical setting are scarce, with a few studies suggesting no association between osteolysis and pain, despite methodological limitations. This study aims to analyze the association between osteolysis and patient clinical complaints, using MAVRIC-SL MRI sequences, as well as to evaluate interobserver and intraobserver agreement on bone resorption.

## MATERIALS AND METHODS

### Study design, participants, and criteria

This was a prospective cross-sectional study of 47 patients (49 joints) seen between March 2019 and August 2020 at the Orthopedic Outpatient Clinic of the Hospital Santa Izabel, in the city of Salvador, Brazil, for postoperative follow-up of knee or hip arthroplasty after complaining of chronic pain, defined as pain persisting for three months or longer^**(^10^)**^. Participation in the study was determined through non-probabilistic sampling of consecutive patients. The study was approved by the Research Ethics Committee of the Hospital Santa Izabel. All procedures were approved by the local institutional review board, and all participants gave written informed consent. Patients for whom MRI was contraindicated would be excluded, as would those with inflammatory arthropathy, those who had previously undergone revision surgery in the joint under study, those with cognitive disorders that would have made it difficult for them to answer the questions on the Western Ontario and McMaster Universities Osteoarthritis Index (WOMAC) questionnaire, those who presented with claustrophobia, and those with marked involuntary movements during the MRI examination, which would have impeded the analysis of the images.

### Clinical evaluation

The patients included in the study were evaluated by the orthopedic team. Sociodemographic characteristics, life habits, and clinical data were collected. After a routine evaluation, all selected patients completed the WOMAC questionnaire to estimate the severity of the disease and were referred for MRI. The WOMAC questionnaire has been validated in several cultures and is considered a reliable measure of clinical outcomes. The Likert variant of the WOMAC, version 3.0, which contains 24 items distributed in three domains (pain, stiffness, and joint function) was used, and each item was scored on a four-point Likert scale, the total score therefore ranging from 0 to 96^**(^11^)**^.

### MRI parameters

The MRI examinations were performed in a 1.5-T scanner (Optima MR 450w with XP, version DV25; GE Healthcare, Waukesha, WI, USA). The protocol consisted of four MAVRIC-SL sequences (GE Healthcare): two fluid-sensitive short-tau inversion recovery (STIR) sequences, in the coronal and axial planes, respectively; one unenhanced axial T1-weighted sequence; and one contrast-enhanced axial T1-weighted sequence. In a subgroup of 20 consecutive patients, one MAVRIC-SL proton density (PD)-weighted sequence, in the sagittal plane, was added, according to the protocols detailed in Tables 1 and 2.

**Table 1:** Protocol of the hip MRI examinations.

Parameter	MRI with MAVRIC-SL sequences				
	Coronal STIR	Axial STIR	Axial T1-weighted	Axial T1-weighted CE	Sagittal PD
TR (ms)	4000-5000	4000-5000	300-700	300-700	3000
TE (ms)	7.4	7.2	7.9	7.9	6.6
TI (ms)	150	150	-	-	-
Echo train (Hz/pixel)	20	20	8	8	20
Slice thickness (mm)	5.0	5.0	6.0	6.0	4.0
Interslice gap (mm)	0.0	0.0	0.0	0.0	0.0
FOV	38 **×** 38	26 **×** 20	32 **×** 28	32 **×** 28	40 **×** 32
Matrix	256 **×** 192	256 **×** 192	320 **×** 224	320 **×** 224	384 **×** 256
Bandwidth	125 kHz	125 kHz	125 kHz	125 kHz	125 kHz
NEX	0.50	0.50	0.50	0.50	0.50

TR, repetition time; TE, echo time; TI, inversion time; FOV, field of view; NEX, number of excitations; CE, contrast-enhanced.

**Table 2:** Protocol of the knee MRI examinations.

Parameter	MRI with MAVRIC-SL sequences				
	Coronal STIR	Axial STIR	Axial T1-weighted	Axial T1-weighted CE	Sagittal PD
TR (ms)	4000-6000	4000-6000	300-700	300-700	3700
TE (ms)	6.8	7.0	7.4	7.4	7.8
TI (ms)	150	150	-	-	-
Echo train (Hz/pixel)	20	20	8	8	20
Slice thickness (mm)	4.0	5.0	5.0	5.0	4.0
Interslice gap (mm)	0.0	0.0	0.0	0.0	0.0
FOV	22 **×** 17	22 **×** 17	22 **×** 17	22 **×** 17	18 **×** 14
Matrix	256 **×** 192	256 **×** 192	320 **×** 192	320 **×** 192	320 **×** 256
Bandwidth	125 kHz	125 kHz	125 kHz	125 kHz	125 kHz
NEX	0.50	0.50	0.50	0.50	0.50

TR, repetition time; TE, echo time; TI, inversion time; FOV, field of view; NEX, number of excitations; CE, contrast-enhanced.

### MRI evaluation

All components of the knee prostheses were evaluated using the radiographic evaluation and scoring system developed by the Knee Society^**(^12^)**^. The hip prostheses were evaluated by DeLee and Charnley zone^**(^13^)**^, for the acetabular component, and by Gruen zone^**(^14^)**^, for the femoral component.

In the analysis of the femoral component of the knee, only four zones were evaluated, that analysis being applicable to both condyles, although each zone was counted only once. For the patellar component, five zones were evaluated in the axial plane. For the tibial component, we evaluated seven zones in the coronal plane and two in the axial plane.

In the acetabular component of the hip, in addition to the DeLee and Charnley zones in the coronal plane, two zones (anterior and posterior) were added in the axial plane. In the coronal and axial planes, the femoral component was analyzed in seven Gruen zones in the medial and lateral interfaces and in six Gruen zones in the anterior and posterior interfaces.

### Variables and biases

**Figure 1:**
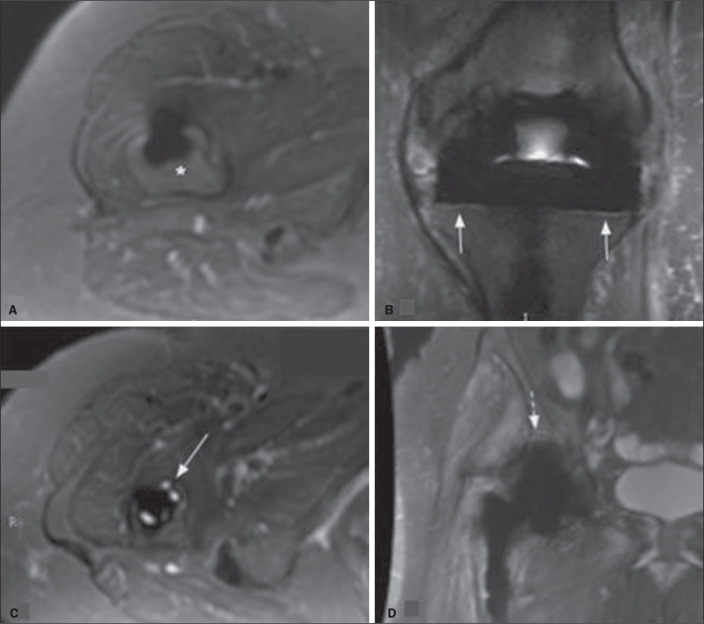
Types of interfaces on MRI (MAVRIC-SL sequences). A: Axial STIR sequence of the hip, showing normal signal intensity (asterisk) at the interface between the prosthesis and the bone marrow. B: Coronal STIR sequence of the knee, showing a thin strip with a slight increase in the signal intensity at the interface between the prosthesis and the tibial plateaus, with low signal intensity at the margin (arrows), consistent with fibrous membrane formation. C: Axial STIR sequence of the hip, showing areas of signal hyperintensity at the anterior interface of the femoral component, together with a sharp edge of low signal intensity, demarcating the boundary with the normal medulla immediately adjacent to it. D: Coronal STIR sequence of the hip, showing magnetic susceptibility artifacts, which made it impossible to properly evaluate the interface in DeLee and Charnley zones I and II (dashed arrow).

The bone-prosthesis or bone-cement interfaces of the prosthesis components were classified as described by Burge et al.^**(^6^)**^: osseointegrated, when there was no change in the STIR signal in the trabecular bone contiguous to the prosthesis or cement (Figure 1A); containing fibrous membrane formation (resorption), when there was a thin layer of hyperintensity at the interface, analogous to the “radiolucent line” on the radiographs, with a thickness up to 2.0 mm, accompanied by a low-signal-intensity sclerotic edge (Figure 1B); and osteolytic, when there was a globular, coarse area of osteolysis (resorption, with a hyperintense signal), more than 2.0 mm thick, at the interface (Figure 1C). When the magnetic susceptibility artifact was severe enough to prevent adequate observation of the interfaces (Figure 1D), the zones were categorized as nondiagnostic.

For the analysis of the main study objective, an evaluation of all interfaces in all components of the prosthesis (a global analysis) was performed. A prosthesis was considered osseointegrated if there were no zones with osteolysis in any of its components and osteolytic if there was at least one osteolytic zone in any one of its components.

Other periarticular findings that were potential confounding factors, such as the source of the pain, were described. A subsample comprising sagittal sequences with PD-weighted imaging was reevaluated, on average, 7.2 months after the initial readings.

Two radiologists, specialists in the imaging of the musculoskeletal system (both with 13 years of experience), reviewed the criteria for bone osteolysis on MRI. In the agreement analysis, osseointegration and resorption zones were defined for each prosthetic component separately. To be considered osseointegrated, a component had to have no areas of resorption on MRI, even if there were nondiagnostic zones. A component was considered to have resorption zones if there was at least one zone of any type of resorption (osteolysis or fibrous membrane formation). The interobserver agreement was analyzed for the two radiologists, who were working independently and were blinded to the patient histories and clinical data. Similarly, as a measure of intraobserver reproducibility, the same diagnosis was described an average of 7.2 months after the first evaluation. The radiologists analyzed the images at workstations, using the Carestream Picture Archiving and Communication System, version 12.0 (Carestream Health, Rochester, NY, USA).

### Sample size calculation and statistical analysis

We calculated that 42 participants would be required in order to obtain a statistical power of 80% in the detection of a difference of 14 points of severity in the WOMAC score, with a type I error of 5%, considering a standard deviation of the WOMAC score of 16 points^**(^15^)**^. On the basis of the MAVRIC-SL findings, the prostheses were divided into two groups: osseointegrated and osteolytic. To identify differences in the mean of the WOMAC clinical score (dependent variable) between the osseointegrated and osteolytic groups, the Mann-Whitney U test was used. Pearson’s chi-square test was used in order to quantify associations between the THA and TKA groups in terms of sociodemographic and clinical variables. In the analysis of confounding factors, the Mann-Whitney U test was employed to identify differences in the mean WOMAC score between the groups with and without other potential pain findings. We used the Wilcoxon signed-rank test to detect differences in the sum of the resorption zones (fibrous membrane formation plus osteolysis) after the addition of the PD-weighted sequences to the original protocol. Cohen’s kappa statistic was used for the analysis of observer agreement, with the following classification^**(^16^)**^: < 0.00 = poor; 0.00-0.20 = slight; 0.21-0.40 = fair; 0.41-0.60 = moderate; 0.61-0.80 = substantial; 0.81-1.00 = almost perfect. Statistical analyses were performed with the Statistical Package for the Social Sciences, version 14.0.1 (SPSS Inc., Chicago, IL, USA). For all tests, values of *p* < 0.05 were considered statistically significant.

## RESULTS

All patients who met the study criteria were included in the study; there were no exclusions. The mean age of the patients was 66 ± 7.2 years. Most (77.5%) of the patients were women. The most common indication for arthroplasty (in 87.8% of the cases) was osteoarthritis. There were no significant differences between the osseointegrated and osteolytic groups in terms of the sociodemographic or clinical variables, except for body mass index, the proportion of patients with overweight or obesity class I being higher in the osteolytic group, as shown in Table 3.

**Table 3:** Sociodemographic and clinical characteristics of the patients, by prosthesis group.

Characteristic	Osseointegrated (n)	Osteolytic (n)	Total n (%)	*P* ^*^
Age (years)				0.49
≤ 59	2	7	9 (18.4)	
> 59	12	28	40 (81.6)	
Gender				0.61
Female	11	27	38 (77.5)	
Male	3	8	11 (22.5)	
Race				0.20
Black	2	10	12 (24.5)	
White	1	7	8 (16.3)	
Indigenous	0	2	2 (4.1)	
Mixed	11	16	27 (55.1)	
Time since surgery				0.16
3-5 months	5	7	12 (24.5)	
6-12 months	1	10	11 (22.5)	
13-18 months	3	3	6 (12.2)	
19-24 months	0	5	5 (10.2)	
> 24 months	5	10	15 (30.6)	
Time since pain onset				0.16
3-5 months	4	11	15 (30.6)	
6-12 months	6	14	20 (40.8)	
13-18 months	3	1	4 (8.2)	
19-24 months	0	5	5 (10.2)	
> 24 months	1	4	5 (10.2)	
Body mass index				**0.032** ^†^
Normal	2	6	8 (16.3)	
Overweight	3	15	18 (36.7)	
Obesity class I	6	14	20 (40.8)	
Obesity class II	3	0	3 (6.2)	
Underlying cause				
Osteoarthritis	14	29	43 (87.8)	0.43
Osteonecrosis	0	1	1 (2.0)	
Trauma	0	2	2 (4.1)	
Other	0	3	3 (6.1)	
Visual analogue scale				0.81
0-2	1	2	3 (6.1)	
3-7	6	12	18 (36.7)	
8-10	7	21	28 (57.2)	

* Pearson’s chi-square test.

† Statistically significant.

The mean time from clinical evaluation to MRI was 26 days (interquartile range, 17-47 days). Our sample included 32 TKAs (65%), 16 THAs (33%) and one partial hip arthroplasty (2%); a total of 101 prosthetic components were evaluated. All prostheses were of the metal-on-polyethylene type (cobalt-chromium alloy with cross-linked polyethylene), and the patellar components were made of polyethylene. All components of the knee prostheses were of the cemented type. Among the hip prostheses, the femoral component was cemented in 10 cases and, in all but one case, the acetabular component was of the uncemented type. In the partial arthroplasty case, the prosthesis was cemented.

**Figure 2:**
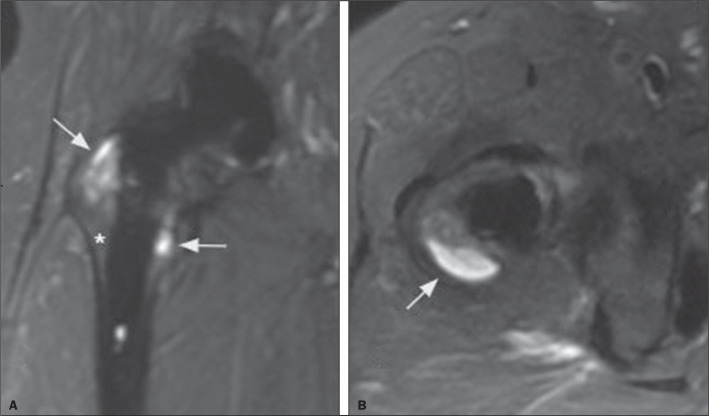
Osteolytic and osseointegrated zones in THA on MRI (MAVRIC-SL). A: Coronal STIR sequence, showing hyperintense interfaces in Gruen zones 1 and 7 (arrows), corresponding to osteolytic zones, and one interface with no signal abnormality in Gruen zone 2 (asterisk), corresponding to an osseointegrated zone. B: Axial STIR sequence, showing a grossly hyperintense interface in Gruen zone 8 (arrow), corresponding to another osteolytic zone.

There was a statistically significant difference between the osseointegrated and osteolytic groups in terms of the mean total WOMAC score, as well as the scores on the stiffness and function domains (Table 4). A total of 742 interface zones were analyzed, and most of them were categorized as osseointegrated. Bone resorption zones accounted for 19.4% of the total, most of the resorption being attributed to areas of osteolysis, as shown in Figure 2. Only a few of the zones evaluated were considered nondiagnostic. Of the 32 knee prostheses evaluated, 15 (46.9%) had at least one zone with osteolysis, which was seen in 11 (64.7%) of the 17 hip prostheses evaluated.

**Table 4:** Clinical severity, by periprosthetic osteolysis.

WOMAC score	Periprosthetic osteolysis		*P* ^*^
	Yes (n = 26)	No (n = 23)	
Total	29.92	19.43	**0.010** ^†^
By domain			
Pain	28.62	20.91	0.059
Stiffness	28.38	21.17	**0.047** ^†^
Function	29.90	19.46	**0.011** ^†^

* Mann-Whitney U test.

† Statistically significant.

Findings in periarticular or local soft tissues were identified in 37.5% of the knees and in 47.1% of the hips (Table 5). However, those findings showed no statistical association with the clinical complaints of the patients, as determined by the WOMAC score (*p* = 0.91). The addition of the sagittal PD-weighted sequence resulted in no significant change in the analysis of the interfaces with the prosthesis (*p* = 0.63).

**Table 5:** Additional findings with potential for pain.

Finding			Joints n (%)
In TKA			
Cement extravasation into soft tissues			1 (3.1)
Pes anserine bursitis			3 (9.4)
Baker cyst (with rupture or loose bodies)			4 (12.5)
Cyst in periarticular soft tissues			1 (3.1)
Microfracture of the bone trabeculae in the medial tibial condyle			1 (3.1)
Deep infrapatellar bursitis			2 (6.2)
Bursitis of the medial collateral ligament			1 (3.1)
Semimembranosus bursitis			1 (3.1)
Limb length discrepancy			3 (9.4)
In THA			
Small fl uid collection in the deep subcutaneous tissue			1 (5.9)
Trochanteric bursitis			3 (17.6)
Cement extravasation into soft parts of the pelvis			1 (5.9)
Pseudotumor			3 (17.6)
Limb length discrepancy			2 (13.3)

The analysis of interobserver agreement showed substantial agreement on both components among the THA cases and for the tibial component among the TKA cases (Table 6). The analysis of intraobserver agreement showed substantial agreement on the femoral component and moderate agreement on the acetabular component among the THA cases, as well as substantial agreement on the femoral and tibial components among the TKA cases (Table 6).

**Table 6:** Interobserver and intraobserver agreement.

Agreement analyzed	Site	Cohen’s kappa	*P* ^*^
Interobserver THA	Acetabulum	0.72	**0.047** ^†^
	Femur	0.77	**0.001** ^†^
TKA	Femur	0.33	0.104
	Tibia	0.72	< **0.001**^†^
	Patella	0.50	0.248
Intraobserver THA	Acetabulum	0.57	**0.031** ^†^
	Femur	0.64	**0.008** ^†^
TKA	Femur	0.76	< **0.001**^†^
	Tibia	0.62	< **0.001**^†^
	Patella	0.50	0.248

* Kappa test.

† Statistically significant.

## DISCUSSION

Although the periosteum is considered the core of bone pain, there is clinical and experimental evidence of periosteal and medullary innervation by afferent nociceptors, which are sensitive to mechanical, chemical, and thermal stimuli^**(^17^)**^. That evidence supports the notion that inflammatory and mechanical changes involved in the process of bone resorption in the medulla adjacent to the prosthesis contribute to the clinical deterioration experienced by patients with osteolytic zones. In the present study, we detected an association between osteolysis in the interfaces and the clinical condition of the patients, especially that related to joint stiffness and function. In a study comparing asymptomatic and symptomatic patients, Chang et al.^**(^18^)**^ analyzed osteolysis on MRI as a predictor of pain and found no such association. Their results could have been influenced by the fact that the images were acquired in conventional fast spin-echo sequences, which are known to suffer from artifacts and have lower sensitivity in the evaluation of the interfaces with prostheses than do MAVRIC-SL sequences. In our study sample, there was no statistically significant difference in the pain domain, although we recognize the possibility that our sample size was insufficient to demonstrate such an association.

Our sample was homogeneous in terms of the clinical characteristics of the patients, except for the body mass index. Ba*s*delio*g*lu^**(^19^)**^, analyzing 588 patients who underwent TKA, found that obesity was one of the most important risk factors for infection and aseptic loosening. However, there is still controversy in the literature regarding the association between body mass index and negative outcomes in the postoperative period after knee arthroplasty^**(^20^)**^.

There are many potential causes of regional pain after arthroplasty^**(^21^)**^. In addition to osteolysis, we found at least one aspect that could be implicated as the cause of the clinical complaint in 40.8% of cases, a potential confounding factor in our study. However, no association was found between those additional findings and the clinical condition of the patients, which highlights the role of osteolysis in the genesis of the clinical complaint after arthroplasty in our sample.

Our findings underscore the excellent performance of MAVRIC-SL sequences in the analysis of periprosthetic interfaces composed of ferromagnetic metal alloy, given that there was adequate visibility in approximately 92% of the hip zones and 99% of the knee zones. Their use has gained prominence in the literature as a means of improving the visualization of the periprosthetic region^**(^5^,^22^)**^.

The protocols for evaluating complications after arthroplasty commonly use PD-weighted sequences because of their excellent tissue contrast (distinguishing tissue from fluid) and clear demonstration of bone resorption at the interfaces^**(^23^)**^. Our study protocol was based on MAVRIC-SL STIR and T1-weighted sequences. However, reassessment in a subsample with the addition of the PD-weighted MAVRIC-SL sequence showed no significant change in the diagnosis of resorption in the prosthesis interfaces in our sample, thus corroborating our initial results.

We observed substantial interobserver agreement in most analyses, which is in keeping with the results obtained by Burge et al.^**(^6^)**^ and Kleeblad et al.^**(^9^)**^, confirming the validity of the method for this type of study. However, we detected no statistical significance in the analysis of interobserver agreement for the femoral and patellar components of the TKAs, because that subsample consisted of a limited number of patients, compromising the interpretation of the kappa statistic. To our knowledge, this is the first study to analyze intraobserver agreement in the evaluation of prosthesis interfaces, demonstrating substantial agreement in most analyses.

Our study has some limitations. First, we did not compare the MRI resorption findings with the surgical findings. In addition, the cement-prosthesis interfaces, which can also be responsible for loosening, were not evaluated, because of the low contrast inherent to these materials on MRI, which could result in reduced sensitivity in the evaluation of loosening^**(^6^,^24^)**^. Furthermore, we did not evaluate some recognized potential sources of pain, such as pain radiating to a THA (from low back pain or knee osteoarthritis), neuropathic metabolic pain, instability without frank displacement, and complex regional pain syndrome^**(^21^)**^. Moreover, infection was not ruled out, especially in the subclinical context. Finally, because our protocol used intravenous contrast, it was not possible to include a control group. Although the study was prospective in essence, we did not evaluate the prosthesis interfaces in asymptomatic patients. Case-control studies (of symptomatic versus asymptomatic patients) using MAVRIC-SL sequences could provide more robust results regarding the association between osteolysis in the interfaces and the clinical condition of patients.

## CONCLUSION

Our preliminary results suggest that periprosthetic osteolysis is associated with clinical complaints and loss of joint function. In the context of chronic post-arthroplasty complaints, a challenging scenario for orthopedists, MRI with MAVRIC-SL sequences could play an important role, identifying these periprosthetic causes of poor outcomes, in a reproducible assessment. However, external validation studies are needed in order to corroborate our findings.
